# Items

**Published:** 1882-08

**Authors:** 


### Items.

---

METEOROLOGICAL DATA.—For the month of June, 1881. H. Hall, Observer, U. S. Signal Station, Atlanta, Ga.

| | | |
|---|---|---|
| BAROMETER | —Mean | 30.013 |
| | Highest | 30.239 on 6th. |
| | Lowest | 29.760 on 1st. |
| | Range | 0.479 |
| THERMOMETER | —Mean | 76.2 |
| | Highest | 93.2 on 26–28 |
| | Lowest | 55.5 on 6th. |
| | Range | 37.7 |
| | Greatest Daily Range | 24.3 on 17th. |
| | Least Daily Range | 10.7 on 1st. |
| Humidity | —Mean | 68.0 |
| Dew-point | —Mean | 64.0 |
| Rainfall | —Total | 3.22 inches. |
| | Greatest Daily | 1.45 on 15th. |
| | Total Number of Days | 13 |
| Wind | —Prevailing Direction, N. W. | |
| | Maximum Velocity, N. | 49 miles. |

The following communication was addressed to Dr. W. S. Elkin, of this city, and he has consented to represent the Medical Department of the University of Pennsylvania as requested. Examinations will be held in his office during the first week in September, at ten o'clock a. m. daily:

PHILADELPHIA, July 11, 1882.

It is the intention of the authorities of the University of Pennsylvania, to arrange for the holding of an admission examination to its Medical Department in cities remote from Philadelphia, in order that students residing at a distance may not be discouraged from applying on account of what would seem a useless expense in coming to Philadelphia in the event of their failure to pass the required examination.

My object in writing to you is to ask you to take charge of the examination in your city.

The plan to be pursued is as follows:

I. The examinations are to be held in the different places at the same time which will be this year during the first week in September.

II. For say two days previous to the examination a short advertisement containing information of the fact shall be put in one or two newspapers of the State or district in which the examination is to be held and if possible some editorial notice secured.

III. The candidate invited by the advertisement shall come to your office at such hours as may be most convenient to you and pass his examination under your direction. This will consist first in the writing of an English essay on any subject the candidate may select, and of at least one page of foolscap paper in length. Second, in answering on paper a set of questions on physics which questions will be mailed to you in advance.

IV. You will then mail the essays and answers to us and we will examine them and notify the candidate of his success or failure. The expenses of advertising, stationery and postage will be paid by the University.

Of course we do not expect a large number to present themselves. In some places there may be one or two or three; in most, perhaps, none. And we trust that the trouble to which you will be put will not be more than you are willing to undertake for the sake of Alma Mater.

You will understand that the examination is only required of those who do not possess a collegiate degree or such certificate as is referred to on page 46 of catalogue which I mail. In order to save you trouble this is stated in the form of advertisement which I enclose.

Will you kindly reply stating whether you are willing to serve? Very truly yours,

JAMES TYSON,
Secretary, Faculty of Medicine.

---

THE British Medical Association was organized July 19, 1832, by a party of about fifty medical men who met for the purpose in the Worcester Infirmary on the invitation of Dr. Charles Hastings. Dr. Edward Johnstone was the first president, and Drs. Hastings and Sheppard were appointed secretaries, and the duty of editing the transactions devolved upon them.

The "Jubilee" is appointed to be celebrated on its fiftieth birthday—continuing four days. It is said that a surplus of $50,000 lies in its treasury; and that the influence of the Association upon the profession, as well as upon the medical legislation of Great Britain, is incalculable.

The *British Medical Journal* is the official organ of the Association—in short is its transactions—and increases its power enormously. The *Journal* was established in 1841, with Jonathan Hutchinson as editor. Earnest Hart, the present editor, succeeded him in 1866.

---

AT Sheffield, (*Med. Press and Circular*), recently, a blacksmith, named Malloy, sought to recover two guineas from a Mr. Spowart and a member of the local police force, as damages for trespass. Mr. Spowart and the constable had arrived at Malloy's house at an appointed time to make a post-mortem examination on the body of his son, on which an inquest had been held, when they found a woman had locked the door and left the dwelling. Entering through a window the police-constable let in the surgeon, who made the post-mortem examination, and hence the action. The Judge held that the defendants went to the house in the execution of a public duty, and although they had committed a trespass not justified by law, yet their conduct, considering the peculiar circumstances of the case, was almost justifiable. Verdict for the plaintiff —one farthing without costs.

---

A WRITER in the *Philadelphia Medical Times,* in an article on "The relations of forests to health-resorts," says: "We have examples of winter forest resorts adapted to the treatment of cases of every description, throughout some of our Southern States, particularly South Carolina and Georgia. From Aiken, South Carolina, all the way to Thomasville, Georgia, at an elevation from five hundred to two thousand feet, there are abundant pine forests.

"Along this extensive wooded region already there are to be found many winter health-stations, among which we may mention Eastman's and *Mt. Airy, near Macon, South Carolina,* and Thomasville, in South Georgia."

How some of our modern writers on climatology and climate-cures do get their geography mixed!

---

DR. WILLIAM A. HAMMOND says (*Med. Gazette*): "My opinion of the new code (of New York) can be expressed in a few words.

"I think it illogical, absurd, sophistical, unsound, unwarranted, untenable, inconclusive, fallacious, specious, evasive, inelegant, heretical, unreasonable, unscientific, narrow-minded, visionary and futile.

"But then, I think the old code was worse, and that no code could be any better."

How is that for voluble descriptive epithets, for orthodoxy and for loyalty to the recognized ethical standard of American physicians?

---

DR. KEDZIE, of the Michigan State Board of Health, is authority for the statement that the addition of borax to the starch with which light fabrics are dressed, will render them uninflammable. One teaspoonful of borax to a pint of mixed starch is the proper proportion. It is claimed that the gauziest and most inflammable textures, when treated in this simple way, are rendered absolutely flame-proof.

---

UNDER a recent act of Congress, Surgeon-General Barnes has been retired, having reached the age of sixty-four years, and the President has nominated Dr. Charles H. Crane, the Assistant Surgeon-General, to fill the vacancy.

Dr. Crane is fifty-seven years old, and has been connected with the army since 1848. His first service was in Mexico as assistant surgeon. The appointment is regarded as a most satisfactory one.

---

MYOPIA in France, according to the recent report of a committee appointed by the government to inquire into the cause of its prevalence, is due to the fact that school books are printed in type too finely cut, and that the paper of the books is white. The committee recommends the use of larger face type, and also advises the substitution of white letters on tinted paper for the ordinary black letters on white paper.

---

THE American Pharmaceutical Association will hold its thirtieth annual session at Niagara Falls, beginning Tuesday, September 12th. It has upon its roll the names of thirteen hundred members—twenty-four of whom are credited to Georgia.

It is hoped that this state will be well and largely represented at the approaching meeting. The attractions, so far as the place of assembling is concerned, at least, could not be greater.

---

DR. MCKEW, of Baltimore, according to a note in the *Maryland Medical Journal*, lived from June until November, a period of five months, on milk alone. During that time he attended the duties of a large practice without impairment of strength or losing a pound of flesh. He consumed about three quarters of sweet milk daily.

---

THE *Detroit Lancet* says: The Baltimore Medical and Surgical Society recently refused admission to an applicant—"a well educated and gentlemanly physician"—for membership "solely upon the ground that he had colored blood in his veins."

*Colored blood!* That is considerate, indeed. There is no physiological nonsense about that expression, and the blackest descendant of Ham would not have his most delicate sensibilities touched by the tender allusion.

---

DR. LEWIS D. FORD and Dr. L. A. Dugas, two of the founders of the Georgia Medical College, at Augusta, and for over fifty years members of its faculty, have resigned. They have been unanimously recommended by the faculty for "Professorships Emeriti" in their respective departments, and acceptance, on their part, is insisted upon by their late colleagues.

---

NOTICE TO GRADUATES OF BELLEVUE HOSPITAL MEDICAL COLLEGE.—A second decennial revision of the catalogue of alumni of this college is being prepared for publication, and we are requested to ask that all graduates will send their present address, at once, on a postal card, to the "Historian of the Alumni Association, Bellevue Hospital Medical College, New York City."

---

WE appreciate the kindly allusions of our esteemed neighbor, the *Virginia Medical Monthly.*

---

THE Medical Department of Harvard University refuses admission to women.

